# Location, Location, Location: Five Facts about Tissue Tropism and Pathogenesis

**DOI:** 10.1371/journal.ppat.1005519

**Published:** 2016-05-26

**Authors:** Laura-Isobel McCall, Jair L. Siqueira-Neto, James H. McKerrow

**Affiliations:** Skaggs School of Pharmacy and Pharmaceutical Sciences, University of California San Diego, La Jolla, California, United States of America; University of Wisconsin Medical School, UNITED STATES

Infectious disease burden remains high [1]. An improved knowledge of host–microorganism interactions is key to delineating disease progression and developing new control and treatment modalities. Microorganisms may be pathogenic (able to cause disease) or non-pathogenic (unable to cause disease). Disease severity is influenced by host and pathogen factors; in particular, pathogens may express different virulence factors that modulate disease severity and progression. Within this context, the interaction of the pathogen with organ and tissue niches is especially important. Tropism refers to the ability of a given pathogen to infect a specific location. Organ or tissue tropism reflects the ability of a given pathogen to infect a specific organ or sets of organs. Some pathogens are broadly tropic, infecting all or most organs, while others are restricted to a given tissue or even to certain tissue niches. From this point of view, the ability of a pathogen to infect specific organs may vary over the course of the disease and could be active, pathogen-mediated, or passive, requiring, for example, a prior skin break or vector bite. Within tissue niches, intracellular pathogens may also preferentially infect specific organelles or intracellular sites. This article will focus on tissue tropism and its relationship to pathogenesis with examples from *Staphylococcus aureus* bacteria, *Trypanosoma brucei* protozoan parasites, and the influenza virus.

## Variations in Tropism

*S*. *aureus* resides as a commensal in the nose and upper respiratory tract of 30% of individuals [2]. However, it also has the ability to cause a range of diseases, from localized skin abscesses to endocarditis, pneumonia, osteomyelitis, or disseminated infection [3]. Human African trypanosomiasis (HAT), caused by *T*. *brucei rhodesiense* and *T*. *brucei gambiense*, progresses through two stages of disease. In the first stage, the parasite remains in the peripheral blood and lymphatic system. The second stage is associated with the parasite crossing into the cerebrospinal fluid and ultimately into the brain parenchyma; however, the timing is still under discussion [4]. Parasites present in the cerebrospinal fluid and/or central nervous system (CNS) are also able to traffic back into the bloodstream [5]. Finally, seasonal influenza viruses mainly infect the upper respiratory tract, while pandemic influenza as well as some highly pathogenic avian influenza viruses (e.g., H5N1) have increased ability to infect the lower respiratory tract [6]. Influenza viruses can also infect extrapulmonary tissues, leading, for example, to conjunctivitis [7].

## Impact of Tropism on Disease Severity

As long as *S*. *aureus* resides as a commensal in the upper respiratory tract, it does not cause any symptoms [2]. In contrast, *S*. *aureus* bacteremia has a 15%–50% case fatality rate. Likewise, mortality from *S*. *aureus* infective endocarditis is 22%–66% [3]. Mortality in HAT is due to neurological symptoms that appear once the parasite enters the CNS, leading to approximately 30,000 deaths per year [8]. Slower progression to stage 2 HAT is associated with longer survival [9]. Seasonal influenza causes up to 500,000 deaths per year worldwide, while influenza pandemics can cause millions of deaths (up to 50 million deaths for the 1918 pandemic) [10]. The H5N1 avian influenza virus has about a 60% case fatality rate [6].

## Impact of Tropism on Disease Transmission

Pathogen location also strongly influences transmission to new hosts. Transmission of *S*. *aureus* from bacteremia appears to involve passage through the gastrointestinal tract followed by fecal spread [11]. In contrast, bacterial colonization on the skin and in the nose may facilitate person-to-person transmission [3]. In the case of *T*. *brucei*, CNS parasites are inaccessible to the tsetse fly vector. The presence of parasites in the bloodstream is essential for transmission [5]. The greater transmissibility of seasonal influenza viruses compared to avian influenza viruses may be due in part to the former’s superior ability to colonize the upper respiratory tract [10,12]. Particles in the upper respiratory tract are moved quickly towards the pharynx by the muco-ciliary escalator, whereas particles in the lower respiratory tract are cleared more slowly [13]. Mutations promoting soft palate infectivity also promoted transmission in a ferret influenza model. This environment may be more suitable to the generation of virus-containing droplets; tissue inflammation at this site may also stimulate sneezing, further enhancing transmissibility [14]. Mathematical modeling supports an association between lower infectivity rates deeper in the respiratory tract and enhanced transmission [13].

## Impact of Tropism on Treatment

Drug tissue penetration varies depending on chemical structure, formulation, and delivery. Treatment choices will therefore be influenced by the sites of infection. For example, *S*. *aureus* abscesses may be treated by incision and drainage alone, or with topical antibiotics. In contrast, bacteremia will require systemic antibiotics, and endocarditis may require surgery [3]. Likewise, infection of certain privileged sites complicates drug delivery. For example, stage 1 HAT is easier to treat than stage 2. Stage 2 drugs must be able to cross the blood–brain barrier and are associated with more severe side effects [4]. Elimination of circulating *T*. *brucei* parasites is insufficient to cure patients; infection relapses in the absence of clearance of CNS parasites [5].

## Techniques to Study Mediators of Tropism

Given the importance of tropism in the various scenarios discussed above, identifying the mediators of disease tropism has garnered considerable interest. Key mediators of disease tropism in staphylococcal infections include host characteristics such as immune status, concurrent infections, or medical procedures [3], while bacterial virulence factors include adhesins, metal acquisition genes, toxins, and immune evasion factors [15]. Blood–brain barrier crossing by *T*. *brucei* involves a combination of host and parasite factors. Many of the host factors promoting invasion are also involved in promoting T cell penetration into the brain parenchyma and in the pathogenesis of other infectious agents. These include TNFα, IFNγ, and CXCL10 [5]. Parasite factors are still poorly characterized but may involve proteases such as brucipain (*T*. *brucei* cathepsin L) [16]. Hemagglutinin receptor binding preference to alpha-2,3-linked versus alpha-2,6-linked sialosaccharides is the major determinant of upper versus lower respiratory tract influenza virus tropism, disease severity, and transmission [10,12].

Microenvironmental conditions surrounding the pathogen will alter virulence factor expression. It is, therefore, essential to study mediators of tropism in situ in models that will replicate disease conditions as much as possible. Human samples may be the best source where accessible, but humanized mouse models may represent a suitable compromise [17]. Intravital microscopy has provided significant insights into in vivo pathogen behavior but is limited in depth [4]. Non-invasive tracking methods using new luminescent markers help produce a dynamic time-resolved understanding of disease progression and lead to the identification of new or underestimated sites of infection [7]. Fluorescent markers can also be used to monitor the movement of an individual bacterium or parasite via photoconvertible markers [18], while functional microbial reporters can showcase in situ changes in conditions or pathogen physiology [19].

“Omics” methodologies are also expanding our understanding of disease tropism. Genomic, transcriptomic, proteomic, and metabolomic comparisons of different pathogen strains with variable tropism are regularly used to identify virulence factors (see for example [20], which showed a relationship between *S*. *aureus* toxicity and the ability to cause invasive disease) [9]. Dual host–pathogen RNA-seq using infected organs can also help describe host and pathogen responses in situ. New combinations of forward genetic screens and “omics” techniques are also being developed, such as high-throughput gene inactivation methodologies (RIT-seq) in *T*. *brucei*. These have been used to identify essential genes during in vitro culture and differentiation [8] but could readily be applied to identify parasite genes involved in host infection and tissue tropism. Finally, the increasing ease of genetic manipulation, including CRISPR/Cas9 technology, facilitates the necessary validation of the factors identified in these large-scale studies.

## Conclusions

Disease prevention, monitoring, and treatment are mainstays of modern medicine. In this review, we highlighted the relationship between tissue tropism and disease severity, transmission, and treatment. Many infectious diseases, including sleeping sickness and staphylococcal infections, still lack effective vaccines. Vaccine development requires an understanding of the immune response required for protection, which is associated in part with the tissues targeted. Likewise, predicting disease progression from initial diagnosis remains a significant challenge. Identifying the site of penetration by infecting microorganisms and the tissues involved may help stratify patients and determine the appropriate course of treatment. This is especially important in the case of *S*. *aureus* infection to determine, for example, whether patients are at risk of disseminated infection.

Overall, our understanding of the mediators of tissue tropism has progressed significantly; however, we cannot yet account for all the factors involved. Indeed, fine-scale differences in host tissue chemistry, metabolism, waste production, and local immune responses are still being identified. Moreover, while we usually know the ultimate tissue niche of a specific pathogen, we often do not understand the paths used to reach that location, as evidenced by the controversy surrounding the timing of brain penetration in *T*. *brucei* infection [4]. We are also beginning to appreciate that tropism is dynamic rather than static. Large-scale application of new technologies should facilitate continuing advances in this field and, ultimately, lead to the discovery of new methods to target these pathogens.
